# Important updates from the AACR virtual meeting: COVID-19 and cancer

**DOI:** 10.2144/fsoa-2020-0132

**Published:** 2020-09-18

**Authors:** Jacob Ripp, Anup Kasi

**Affiliations:** 1 Department of Internal Medicine, University of Kansas Medical Center, 3901 Rainbow Boulevard, Kansas City, KS 66160, USA; 2 Division of Medical Oncology, University of Kansas Medical Center, 2650 Shawnee Mission Parkway, Westwood, KS 66205, USA

**Keywords:** cancer, COVID-19, cytokine release syndrome, oncology, vaccine

## Abstract

In this article, we present the pivotal abstracts presented at the ‘AACR Virtual Meeting: COVID-19 and Cancer’ held in July 2020. Much is unknown regarding the effect of COVID-19 on cancer patients, and this conference presented important updates in therapeutics and epidemiology. Specifically, cancer therapeutics have been hypothesized to treat cytokine release syndrome in patients with COVID-19, and the JAK1/2 inhibitor, ruxolitinib, is currently being used in a Phase III trial to assess its efficacy. Additionally, similar to other studies, morbidity and mortality in patients with COVID-19 and cancer appear to be worse in older patients and in males. We also present various COVID-19 data related to breast cancer, lung cancer, hematologic malignancies, melanoma and prostate cancer.

The American Association for Cancer Research (AACR) hosted a meeting during the Coronavirus disease 2019 (COVID-19) pandemic. ‘AACR Virtual Meeting: COVID-19 and Cancer’ is one of the first major scientific meetings discussing the latest basic, clinical and epidemiological data related to COVID-19 and cancer. This meeting was held virtually on July 2020, and 79 total abstracts were presented. In this report, we discuss the most relevant abstracts describing the impact of COVID-19 on cancer.

## Discussion

### Therapeutics to treat cytokine release syndrome

#### RUXCOVID: A Phase III, randomized, placebo-controlled study evaluating the efficacy & safety of ruxolitinib in patients with COVID-19–associated cytokine (NCT04362137)

Cytokine release syndrome (CRS) has been implicated in many patients with severe COVID-19 pneumonia. Unfortunately, treatments for CRS are scarce. Therapeutics used for cancer-directed treatments that produce immunomodulatory effects may be an effective treatment option. In this Phase III, randomized, placebo-controlled study, patients in the experimental group will be given ruxolitinib – a JAK1/2 inhibitor approved to treat myelofibrosis, among other diseases – in addition to standard of care therapy. The target enrollment is 402 patients [1].

#### Expanded access trial of tocilizumab in COVID-19 positive hospitalized cancer patients (NCT04370834)

Phase III trials are currently testing tocilizumab, an IL-6 receptor antibody used to treat CRS in chimeric antigen receptor T-cell therapy patients, in patients with severe COVID-19 who develop CRS (NCT04370834). Due to exclusion criteria, cancer patients may not be eligible for enrollment in pivotal COVID-19 trials. This is a multicenter, single-arm, open-label, expanded-access treatment study to allow cancer patients with severe COVID-19 access to tocilizumab. The trial is currently enrolling [2].

### Therapeutics to treat COVID-19

#### Assessment of COVID-19 vaccine candidates: prediction & validation of 174 SARS-CoV-2 epitopes

Vaccine development for COVID-19 has gained considerable attention given the acute need for prevention. Stimulating an effective immune response is contingent on the presentation of epitopes to T cells. Due to the size of the COVID-19 virus, testing all of the peptides is not a feasible solution. Instead, HLA-binding prediction tools are needed to identify possible peptides to test. Pracher and colleagues tested 19 HLA-binding prediction tools and found 777 peptides thought to be good binders across 11 MHC alleles [3]. Overall, they found 174 severe acute respiratory syndrome coronavirus 2 (SARS-CoV-2) epitopes with high prediction-binding scores that could bind to 11 MHC alleles. This could assist in the development of an effective vaccine against COVID-19.

#### SARS-CoV-2 multiepitope vaccine constructs designed to drive long-term immunity in the majority of the population

Vaccines against COVID-19 must produce a safe immunogenic response based upon the correct selection of epitopes. These epitopes must maximize immunogenicity and minimize antigenic load that could lead to increased infection, autoimmunity or an intolerable immune reaction. Yarmarkovich and colleagues have created an immunogenicity map for COVID-19 to generate 11 vaccine constructs designed for long-term immunity. These constructs are currently undergoing testing in mice expressing human HLA-A2 [4].

#### Low-dose whole-lung radiation for COVID-19 pneumonia: planned day-7 interim analysis of a registered clinical trial

One of the mediators of CRS are lymphocytes, which happen to be very sensitive to ionizing radiation. In the RESCUE 1–19 trial, nine candidates who were hospitalized and oxygen dependent with COVID-19 pneumonia were evaluated to receive low-dose radiation therapy (LD-RT) with a single-fraction dose of 1.5 Gy to the bilateral lungs with the primary end point of safety. One patient died before enrollment, one patient did not have severe enough disease and two were intubated. Five patients received LD-RT. Four out of the five patients clinically recovered without evidence of COVID symptom exacerbation. LD-RT appears safe and merits further investigation in the treatment of severe COVID-19 CRS [5].

### Epidemiology and outcomes of COVID-19 in cancer patients

The incidence of COVID-19 in cancer patients, especially those undergoing cancer-directed treatment is unknown. It is also unknown whether cancer patients have higher incidences or higher mortality from COVID-19 infections than general populations. These abstracts provide data on patients with cancer who were infected with COVID-19.

#### Incidence of SARS-CoV-2 infection in cancer patients undergoing active treatment

This is a study of 692 cancer patients in the Andalusian community in Spain [6]. Overall, nine out of 692 patients were infected with COVID-19 with a cumulative incidence of 1.3% (95% CI: 0.384–2.217), and 22% of these patients died after developing the infection. They found that advanced age (p = 0.011), admission within 3 months of COVID-19 diagnosis (p = 0.031) and active chemotherapy treatment (p = 0.003) were associated with an increased risk of COVID-19 infection in cancer patients. They conclude that while the incidence of COVID-19 in cancer patients is low, the mortality is high.

#### One piece of the jigsaw for the cancer recovery strategy: prevalence of COVID-19 in patients with cancer

In this study at a cancer center in the UK, 654/2647 patients (25%) attending outpatient clinics or receiving chemotherapy or radiotherapy were swabbed for COVID-19 [7]. Nine patients out of 654 (1.38%) were positive for the virus. During this time, 0.27% of the community were infected with COVID-19 (95% CI: 0.17–0.41). However, for those in healthcare roles, the estimated infection rate was 1.33% (95% CI: 0.39–3.28).

#### Outcomes of older cancer patients infected with COVID-19 at Gustave Roussy Cancer Center

In this study from France, 137 cancer patients were diagnosed with COVID-19, with 36 patients (26%) 70 years or older [8]. They found that age was not associated with clinical worsening (hazard ratio: 1.157; 95% CI: 0.55–2.42; p = 0.07). However, age was associated with worse overall survival (hazard ratio: 2.45; 95% CI: 1.02–5.92; p = 0.0463). They conclude that older patients with cancer and diagnosed with COVID-19 had the same rate of worsening, but worse overall survival than younger patients.

#### A prospective testing SARS-CoV-2/COVID-19 in cancer patients with antitumor treatment

In this study from Kansas, 507 patients with cancer were enrolled with the goal of determining COVID-19 status in patients prior to starting anticancer treatment [9]. The median age was 65, and 110 patients (22%) were 75 years or older. Zero patients out of 507 tested positive for COVID-19. They conclude that the prevalence of COVID-19 in asymptomatic cancer patients is low.

#### COVID-Cancer Huil study: registry of oncologic patients diagnosed with COVID-19 in Infanta Leonor University Hospital in Madrid, Spain

This study from Spain reviewed the records of 2216 patients who were hospitalized and found that 85 patients (3.8%) also had a cancer diagnosis [10]. Cancer patients diagnosed with COVID-19 had a significantly higher mortality rate than noncancer patients with COVID-19 (40/85 cancer patients vs 260/2131 in general ward; p < 0.001). The median age was 76 years old and the 50/85 (58.8%) were male. Older patients with cancer also had significantly higher mortality than younger patients (79.5 vs 73; p = 0.03) and in patients with metastatic disease (20/32 metastatic vs 20/53 nonmetastatic patients; p = 0.02).

#### Impact of cancer history on outcomes among hospitalized COVID-19 patients: a case–control analysis

In this study from Boston, 162 cancer patients were diagnosed with COVID-19 and matched 1:2 by age, sex, race and admission date with controls without a cancer history [11]. They found that the rate of mortality or discharge to hospice were similar between patients with cancer and patients without cancer (univariable odds ration [OR]: 1.15, 95% CI: 0.73–1.82; multivariable OR: 1.54, 95% CI: 0.90–2.65). In patients with cancer, patients who had evidence of metastatic disease or were receiving cancer directed therapy within the last 6 months (n = 74, 46%) did not have higher rates of death or discharge to hospice than patients with no evidence of disease and no cancer directed therapy within the last 6 months (univariable OR: 1.37, 95% CI: 0.68–2.75; multivariable OR: 1.77, 95% CI: 0.80–3.93).

#### Preliminary analysis of factors associated with COVID-19-related disease severity in cancer patients: University of Kansas Cancer Center experience

This study from Kansas identified 40 patients with a cancer diagnosis who were diagnosed with COVID-19 [12]. They found that patients with cancer and COVID-19 who had severe illness were significantly older than those with mild to moderate illness (median age 67.5 vs median age 56.5; p = 0.02). They also found that sex, race and ethnicity, obesity, functional status, cancer type, active cancer treatment and recent surgery were not associated with disease severity. They also found that cancer patients who were symptomatic had significant higher ferritin levels (n = 17) than asymptomatic patients (n = 2) (median ferritin 422 vs 68.5; p = 0.04).

#### Sex bias in COVID-19-associated illness severity & mortality in cancer patients: a systematic review & meta-analysis

There has been growing evidence that COVID-19 infection has significantly higher mortality in males than females. It is unknown if this finding exists in patients with cancer and COVID-19 infection. This study reviewed 2764 patients across nine studies of cancer patients with COVID-19 and found that pooled overall risks for the composite outcome of severe illness and death attributable to COVID-19 was 1.68 (95% CI: 1.27–2.24), death was 1.98 (95% CI: 1.21–3.26) and severe illness was 1.48 (95% CI: 1.05–2.10), all disfavoring males [13]. They conclude that this unique finding of male sex bias and higher rates of COVID-19 morbidity and mortality extends to cancer patients as well.

### COVID-19 impact on cancer treatment

Studies of the impact of COVID-19 on specific cancers is also scarce. These abstracts reveal important COVID-19 findings in breast cancer, lung cancer, multiple myeloma, melanoma and prostate cancer.

#### Breast

##### Impact of CDK 4/6 inhibitor withdrawal or dose adjustment on COVID-19 incidence in hormone-positive/HER2-negative metastatic breast cancer patients during the pandemic

The standard of care in patients with hormone-positive and HER2 negative metastatic breast cancer (mBC) is hormone therapy in combination with CDK 4/6is. However, one of the main toxicities of CDK 4/6is is neutropenia which may impact patients with mBC diagnosed with COVID-19. This study from Spain evaluated whether dose-reducing or withdrawing CDK 4/6is led to a lower incidence of COVID-19 infection [14]. In total, 6/78 patients (7.7%) with mBC on hormone and CDK 4/6i therapy were diagnosed with COVID-19. Five out of 39 patients diagnosed with COVID-19 were receiving normal regimens of CDK 4/6i therapy while one out of 39 patients who were receiving dose-reduced or discontinued CDK 4/6i therapy was diagnosed with COVID-19. While not significant, there was a trend toward reduced COVID-19 infection in patients with dose-reduced or withdrawn CDK 4/6i therapy.

##### Strong decline of newly diagnosed gynecologic & breast cancers during the COVID-19 pandemic: a retrospective analysis from Innsbruck, Austria

In mid-March, most regions around the world instituted a ‘lockdown’ in order to contain the spread. This also resulted in severe restrictions in access to the healthcare system as well, and patients faced barriers to access routine care, which includes preventive cancer screening. This study from Austria performed a retrospective analysis of newly diagnosed gynecologic and breast cancers at their center from January to May 2019 compared with January–May 2020 [15]. They found that there was a slight increase of new cancer diagnoses in January and February 2020 compared with the same months in 2019 (13% increase), but a strong decline during the lockdown period (-45% in March 2020 vs March 2019, -63% in April 2020 vs April 2019 and -44% in May 2020 vs May 2019).

#### Lung

##### Changes in lung cancer treatment as a result of the COVID-19 pandemic: a prospective observational study

As COVID-19 is a primarily respiratory disease, there is concern that patients with lung cancer may be particularly vulnerable, and it is unclear whether treatment changes should be made for these patients. This study from Canada evaluated 275 patients with lung cancer with 211 patients receiving active treatment [16]. Of patients with lung cancer receiving active treatment, 121 (57%) experienced at least one change in their treatment plan due to the COVID-19 pandemic. Most of the changes involved either delay (n = 48; 39.7%) or cessation in palliative treatment (n = 18; 14.9%). There were also changes in dosing and schedule for many patients (n = 32; 26.4%). They conclude that altering treatment plans for patients with lung cancer may not be indicated as active cancer treatment is not associated with increased complications from COVID-19.

##### Impact of COVID-19 in continuity of cancer treatment for lung cancer patients

This study from Spain analyzed 242 patients with lung cancer receiving active treatment and found that 125 patients (52%) experienced treatment delay or discontinuation as a result of the COVID-19 pandemic [17]. Of the 32 patients receiving therapy on clinical trials, no treatment delays were experienced by any patients.

#### Hematologic malignancies

##### Outcomes of patients with multiple myeloma with COVID-19 infection

Much of the data regarding COVID-19 and cancer patients focuses on patients with solid tumors. However, patients with hematologic malignancies may be especially vulnerable to COVID-19. This study from New Orleans reviewed 15 patients with multiple myeloma who were diagnosed with COVID-19 [18]. Eleven patients out of 15 were on active treatment, and six (40%) out of the 15 patients died. Additionally, patients older than 65 were found to be at increased risk of death compared with patients less than 65 (p = 0.011). Notably, four out of 15 patients had a history of autologous stem cell transplant, and this was not associated with increased mortality (p = 0.103).

#### Melanoma

##### COVID-19 in melanoma patients: Spanish register

Patients with melanoma have largely been excluded from studies evaluating the impact of COVID-19 on cancer patients. This study from Spain evaluated 64 patients with melanoma who were diagnosed with COVID-19 [19]. They found that the median age of patients who died from COVID-19 was 74 years (range: 49–91) while the age for those who were cured was 64 years (range: 6–95). They also found that 85% of patients who died were male while only 62% of those who were cured were male. They conclude that the risk of death in melanoma from COVID-19 is higher in older patients and in males, but similar according to tumor stage and melanoma therapy.

#### Prostate

##### Prostate cancer & COVID-19: impact of hormonal therapy on severe outcomes

This study from New Orleans sought to identify whether hormonal treatment in patients with prostate cancer and diagnosed with COVID-19 is associated with severe outcomes [20]. They identified 56 patients with prostate cancer who were diagnosed with COVID-19. Of these patients, 15 (26.8%) were on hormonal treatment received within 90 days of their COVID-19 diagnosis. They found that 33 (58.9%) patients required hospitalization, ten (17.9%) required admission to the ICU and 13 (23.2%) died. They found no statistical difference in severe outcomes between patients receiving hormonal therapy and those who did not receive hormonal therapy.

## Conclusion

Utilization of cancer therapies to treat CRS in patients diagnosed with COVID-19 offers promise, and there is currently a Phase III study underway using ruxolitinib, a JAK1/2 inhibitor used to treat myelofibrosis. Consistent with prior studies, several abstracts reported worse morbidity and mortality in older patients and in male patients with COVID-19 and cancer.
